# Formation of Thermally Stable Bulk Heterojunction by Reducing the Polymer and Fullerene Intermixing

**DOI:** 10.1038/s41598-017-09167-4

**Published:** 2017-08-29

**Authors:** Yoonhee Jang, Yun Ju Cho, Minjung Kim, Jeesoo Seok, Hyungju Ahn, Kyungkon Kim

**Affiliations:** 1 Department of Chemistry and Nano Science, Ewha Womans University, Seoul, South Korea; 2 Pohang Accelerator Laboratory, Pohang University of Science and Technology, Pohang, 37673 Korea

## Abstract

A morphologically stable bulk heterojunction (BHJ) with a large heterojunction area is prepared by reducing the portion of the small band gap polymer (PTB7) and fullerene intermixture through a sequential deposition (SqD) of the nanostructured PTB7 and the fullerene layer. The nanostructured PTB7 layer is prepared using a ternary solvent composed of chlorobenzene, 1,8-diiodooctane (DIO) and 1-chloronaphthalene (1-CN). Adding DIO and 1-CN enhances the ordering of PTB7 chains and results in a nanostructured polymer surface. The grazing incidence X-ray diffraction results reveal that the SqD of the nanostructured PTB7 and fullerene layers forms the BHJ with little intermixing between the polymer and the fullerene domains compared to the BHJ formed by the deposition of the blended PTB7 and fullerene solution (BSD). The OPV utilizing the SqD processed BHJ (SqD-OPV) exhibits a power conversion efficiency (PCE) of 7.43%, which is similar to that when the BSD processed BHJ (BSD-OPV) is utilized. Furthermore, the SqD-OPV exhibits an excellent thermal stability. The SqD-OPV maintains its initial PCE even after thermal annealing at 140 °C for 10 days, whereas the BSD-OPV maintains 78% of its initial efficiency under the same condition.

## Introduction

The bulk heterojunction (BHJ) is the most widely used photo-active layer system for organic photovoltaic (OPV) devices^1–3^. The BHJ is mostly formed by the blended solution deposition (BSD), where a BHJ is prepared by depositing a blended solution of electron donating polymer and electron accepting organic semiconductors. The fullerene derivatives have been used mainly as the electron accepting organic semiconductors. Nowadays, many efficient non-fullerene acceptors have been reported to achieve excellent solar cell performance in the BHJ OPV^4^. The BSD process is effective in forming the heterojunction with a large surface area. However, delicate controls of the processing conditions (e.g., donor to acceptor ratio, amount of processing additives, types of solvents, processing temperature, and additional annealing process) are required to maximize the solar cell performance by inducing the nano-scale phase separation of the donor and the acceptor materials^5^.

An alternative method of forming the BHJ through sequential deposition (SqD) has recently been reported by several researchers^6–12^. The SqD process is more flexible in finding an optimized condition because the system can be optimized by independently controlling the thickness of each layer. However, finding a method of enhancing the heterojunction area and a proper solution for the top-layer that does not dissolve the bottom-layer are required to achieve efficient SqD processed OPVs (SqD-OPV). Yang *et al*. claimed that the SqD-OPV based on P3HT/PCBM had the potential to show a better performance than a BSD processed OPV (BSD-OPV) because of a significant reduction in the bimolecular charge recombination^5^. The Schwartz group found that the BHJ morphology formed by the SqD process was almost identical to that formed by the BSD process and could be controlled using tailored semi-orthogonal solvent blends^7^.

In terms of the BHJ morphology, pure polymer and fullerene domains and polymer and fullerene intermixture were reported to co-exist in the BHJ prepared by the BSD^13–16^. The intermixture domain may cause the morphological instability of the BHJ processed by the BSD^16^.

Meanwhile, the polymer crystallinity was not disturbed by the SqD of fullerene, which would help in enhancing the morphological stability^12^. However, as the BSD process does, the SqD-OPVs require an additional thermal annealing step to obtain a large heterojunction area because of the ineffective penetration of fullerenes into the polymer layer. The post thermal annealing process might form polymer and fullerene intermixtures.

Therefore, it would be desirable for SqD-OPV that the crystalline and nanostructured polymer surface is preformed and utilized to form a large-area heterojunction with fullerene without an additional thermal annealing step. We chose the poly[4,8-bis[(2-ethylhexyl)oxy]benzo[1,2-b:4,5-b′]dithiophene-2,6-diyl][3-fluoro-2-[(2-thylhexyl)carbonyl]thieno[3,4-b]-thiophenediyl] (PTB7) as the electron donating polymer because it showed an efficient solar cell performance when it was fabricated by the BSD process^17^.

In this study, we have developed a method of increasing the surface area of (PTB7) film by roughening the surface of the polymer in nanoscale using a ternary solvent containing two different polymer chain ordering agents (OA). The roughened surface of the PTB7 film prepared by the ternary solvent was utilized to construct a BHJ with fullerene without a thermal annealing step via the SqD process. The SqD-OPV showed superior solar cell performance compared to the SqD-OPV prepared by a single solvent. Furthermore, the SqD-OPV performance was comparable to that of the BSD-OPV. The thermal stability of SqD-OPV was also significantly superior to that of the BSD-OPV.

## Results and Discussions

The PTB7 bottom layer surface must first be roughened to construct a BHJ with a large heterojunction area through SqD. This was possible by enhancing the ordering of polymer chains because the amorphous polymer formed a smooth surface, whereas the crystalline polymer had a rough surface.

A small amount of additive with a lower vapor pressure and a little solubility for the polymer was added to the host solvent to enhance the ordering of polymer chains. The low-vapor pressure additive would reduce the vapor pressure of the solvent and allow the polymer chains more time for ordering. In addition, the remaining additive would promote the polymer precipitation after the host solvent evaporation, thereby leading to the formation of ordered polymer domains. This would be more effective in the SqD process than in the BSD process. In the BSD process, bulky fullerenes that forms a single layer with polymer will hinder the polymer ordering during the film formation.

In this study, we have developed a ternary solvent composed of chlorobenzene (CB), 1,8-diiodooctane (DIO), and 1-chloronaphthalene (1-CN) to enhance the ordering and roughness of the polymer bottom layer. CB was used as the main solvent for dissolving PTB7. DIO and 1-CN were used as polymer chain ordering agents (OA). DIO was chosen as one of the OAs for PTB7 because of its negligible vapor pressure and because PTB7 was insoluble in DIO. DIO has been used as a processing additive to fabricate the BHJ through the BSD process^18, 19^. Beaujuge *et al*. stated that favorable nano-scale morphologies could be formed from the solution containing a low vapor pressure additive^20^. 1-CN was utilized for the ternary solvent system to further enhance the ordering of the polymer chains. The planar π-conjugated structure of 1-CN was expected to assist the ordering of PTB7. In the cooling crystallization process, a high supersaturation usually results in the creation of a higher number of nucleates and a smaller size compared to low supersaturation. Similarly, in the evaporation-induced crystallization process, the evaporation from high to low supersaturation usually results in a larger number of crystals with a smaller size. 1-CN had a higher boiling point than CB and specific π–π interactions with the polymer main chain. Hence, PTB7 dissolved in the ternary solvent would form a higher saturation than that dissolved in the solvent containing only CB, leading to a larger number of nano-sized domains on the PTB7 film surface and resulting in an enhanced surface area.

The binary solvent consisting of 95 vol% CB and 5 vol% of DIO was first prepared. The ternary solvents were then prepared by varying the ratio of the binary solvent to 1-CN. Table 1 shows the root mean square roughness (*R*_q_) values of the films obtained by atomic force microscopy (AFM). The *R*_q_ value of the PTB7 films increased with the increase of the 1-CN concentration and reached maximum (1.59 nm) at the binary solvent:1-CN ratio of 94 vol%:4 vol%. This value decreased after further increase in the 1-CN portion. It is thought that when the concentration of the 1-CN was further increased, the domains of PTB7 were formed at lower supersaturated state, and consequently the surface roughness of the film was reduced.

**Table 1:** Root mean square roughness (*R*_q_) values of the PTB7 films prepared by various ternary solvents.

vol% of the binary solvent (95 vol% CB and 5 vol% DIO)	vol% of 1-CN	*R*_*q*_ value
100	0	1.46
97	3	1.17
94	6	1.59
88	12	0.82
50	50	0.536
0	100	0.425

**Figure 1:**
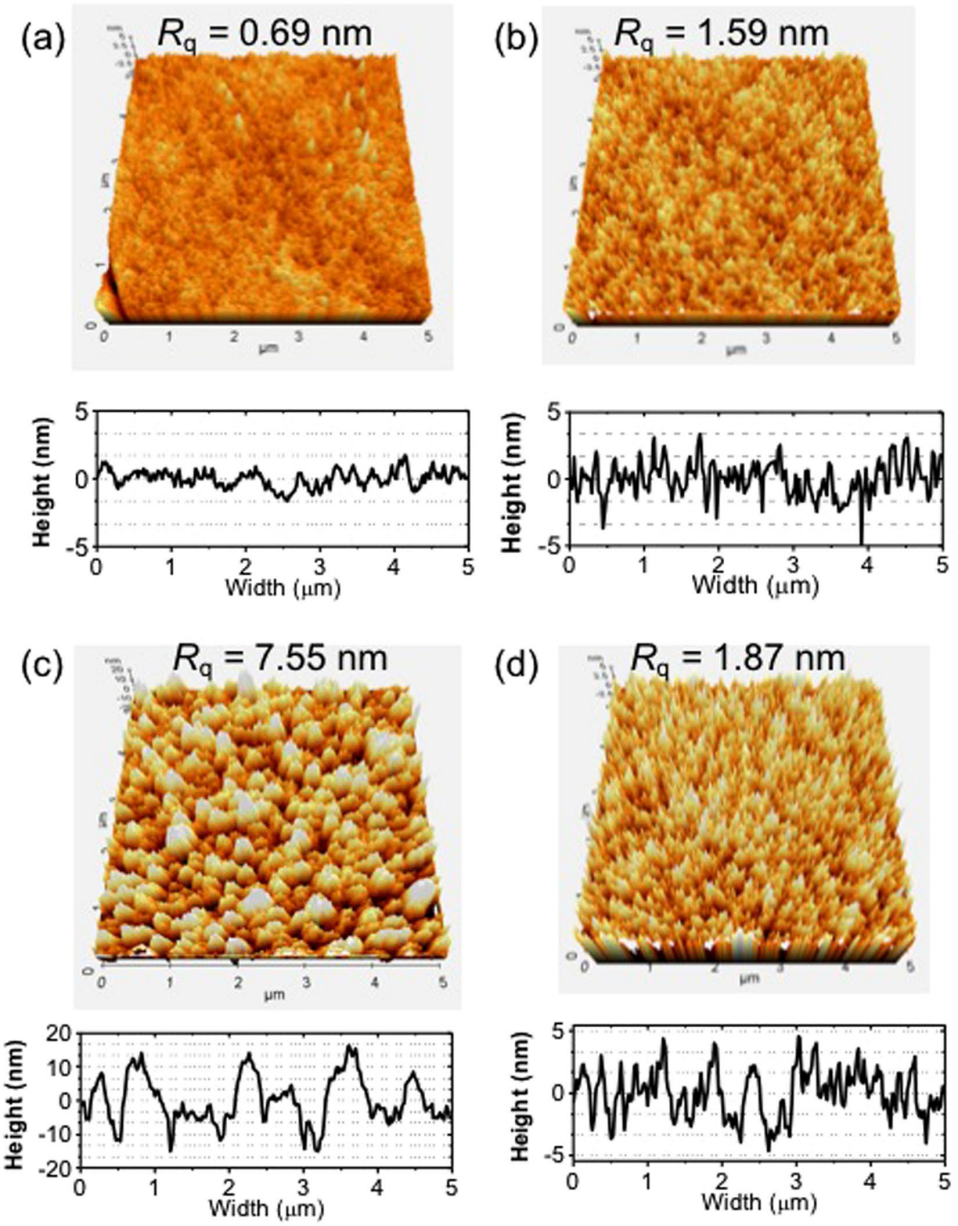
Surface morphologies and height profiles of the as prepared PTB7 bottom-layer and the PTB7 bottom-layer after removal of the PCBM from the PTB7/PCBM films. (**a**) PTB7(S), (**b**) PTB7(T), (**c**) PTB7(S)/[PC_70_BM], and (**d**) PTB7(T)/[PC_70_BM]. All images are obtained by AFM in the tapping mode with a scale of 5 × 5 μm^2^.

Figure 1 shows a comparison of the topology and height profiles of the PTB7 films prepared by the single and ternary solvents denoted as PTB7(S) and PTB7 (T), respectively. The root mean square roughness (*R*_q_) value of PTB7(T) (1.59 nm) was 2.3 times greater than that of PTB7(S) (0.69 nm).

**Figure 2:**
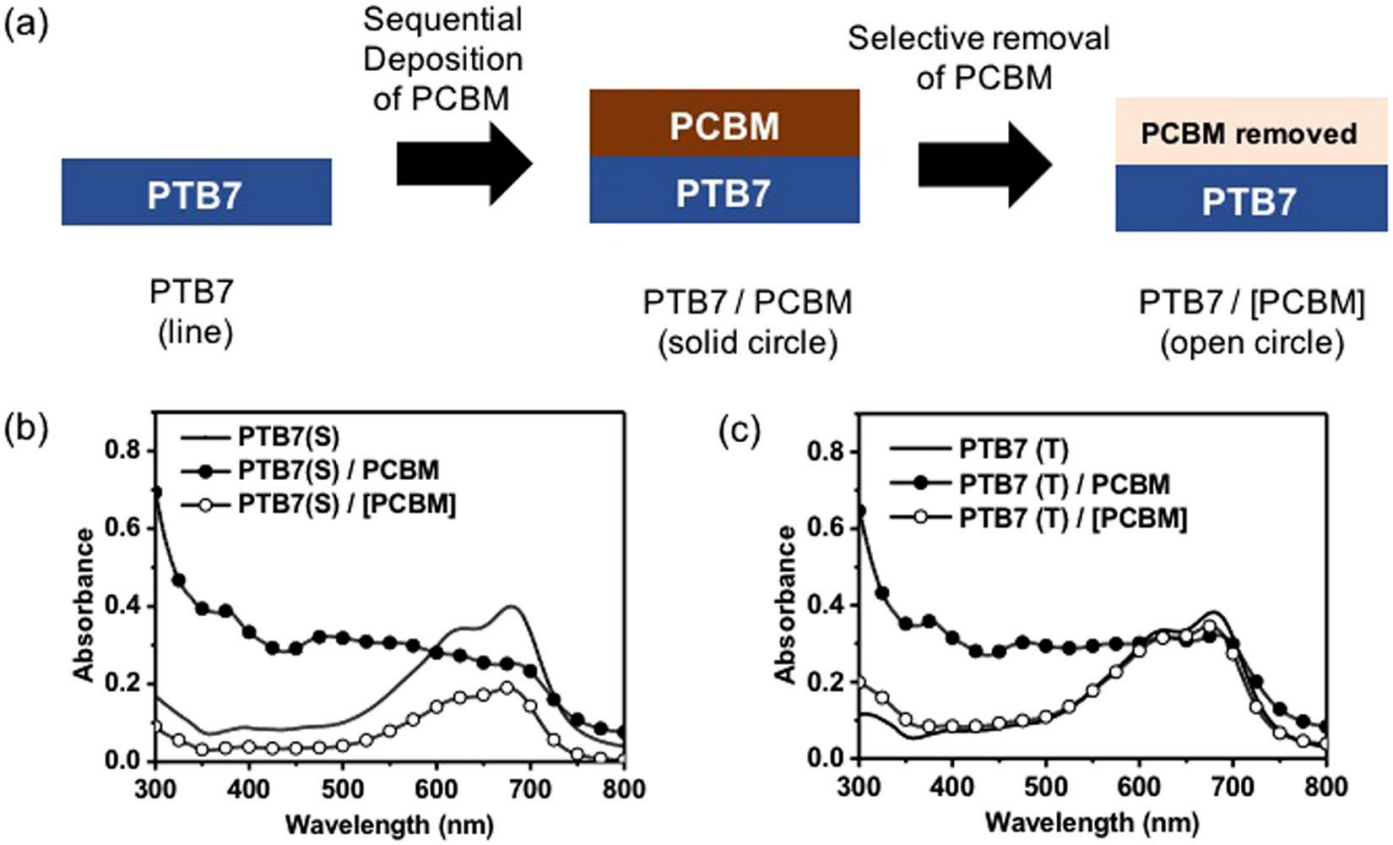
(**a**) Film preparation procedure by the SqD. UV–visible absorption spectra of the PTB7 films prepared by (**b**) single and (**c**) ternary solvents. The solid line, solid circle symbol, and open circle symbol represent the as-prepared PTB7, PTB7/PCBM, and PTB7 films, respectively, after removal of the PCBM from the PTB7/PCBM film.

The PTB7 films with a roughened surface were applied to construct the bulk heterojunction with PCBM through the SqD process. The PCBM was dissolved in the solvent consisting of 96 vol% dichloromethane (DCM) and 4 vol% diiodomethane (DIM) to prepare the PCBM solution. The DIM helped in the conformal coating of the PC_70_BM layer on the PTB7 bottom layer by reducing the vapor pressure of the solution. We investigated whether the PCBM solvent dissolved the PTB7 bottom layer by spin-coating the PCBM solvent onto the PTB7 layer. Figure 2 shows the absorption intensity change for the PTB7 films before and after the PC_70_BM layer deposition. The black lines in Fig. 2(b) and (c) denote the absorption spectra of the as-prepared PTB7(S) and PTB7(T) films (film ‘PTB7’ in Fig. 2(a)), respectively. All the films showed a similar absorption intensity, regardless of the solvent types. The similar absorption intensity indicated that the PTB7 film thicknesses were not affected by the addition of 1-CN or DIO. Subsequently, the PCBM solution was spin coated on the PTB7 bottom layer (film ‘PTB7/PCBM’ in Fig. 2(a)). Comparing the absorption spectrum with PTB7, PTB7/PCBM showed an increased absorption (solid circle in Fig. 2(b) and (c)) at the wavelength corresponding to the PCBM absorption. The PCBM top-layer was selectively removed using diiodomethane (DIM), which was a good solvent for PC_70_BM, and a non-solvent for PTB7 (film ‘PTB7/[PC_70_BM]’ in Fig. 2(a)) to investigate the change in the PTB7 bottom layer thickness after spin coating with the PCBM solution. The open circles in Fig. 2(b) and (c) represent the absorption spectrum of the corresponding PTB7 bottom layer after removal of the PCBM top layer. The absorption intensity of PTB7(S) decreased from 0.40 to 0.19 spectra as PTB7(S) became PTB7(S)/[PCBM] (Fig. 2(b)), indicating that ~52% of PTB7(S) was removed by the PCBM solution. The absorption spectrum of PTB(S) washed by the pure DCM solvent is shown in Figure S1(a) in Supporting Information. As shown, about 90% of PTB(S) was removed by the DCM solvent. This result contrasted with PTB7(T), which maintained 90.5% of its initial thickness. This difference was thought to be caused by the difference in the ordering of the polymer chains. The domains of the ordered PTB7 chains would prevent the PTB7 dissolution by blocking the DCM penetration. According to the absorption spectrum results, the ordered domain of PTB7 would not be easily removed by the PC_70_BM solvent and would maintain its morphology even after the SqD process. This expectation was confirmed by the glazing incident X-ray diffraction (GIXRD) results and will be discussed later.

The heterojunction morphology formed between PTB7 and PCBM was investigated by scanning the PTB7 film topology after PCBM removal. Figure 1(c) and (d) show the surface morphology of the PTB7(S) and PTB7(T) bottom layer, respectively, after the PCBM layer removal. They are denoted as PTB7(S)/[PCBM] and PTB7(T)/[PCBM] in Fig. 2(a), respectively.

A significant difference was observed in the surface morphology between PTB7(s) (Fig. 1(a)) and PTB7(S)/[PCBM] (Fig. 1(c)). The *R*_q_ value of PTB7(s) was greatly enhanced from 0.69 nm to 7.55 nm after the PCBM removal from PTB7(S)/[PCBM]. However, the surface morphology of PTB7(T)/[PCBM] ((Fig. 1(d)) indicated that the film morphology of PTB7(T) (Fig. 1(b)) was relatively well preserved even after the SqD process.

These surface morphology changes were attributed to the partial dissolution of the less ordered PTB7 chains by the PC_70_BM solution during the SqD process. The dissolution amount would depend on the chain ordering of PTB7, which was coincidental with the absorption intensity difference between the PTB7 and PTB7/[PCBM] films in Fig. 2. PTB7(S) was expected to have a lower polymer chain ordering. Hence, a large amount of PTB7 would be dissolved by the DCM (PCBM solvent), followed by the instant formation of the blended solution of PTB7 and PCBM. Furthermore, large PTB7 and PCBM domains would be formed during the DCM evaporation because of the low solubility of PTB7 in DCM. Meanwhile, a slight diffusion of PCBM into the PTB7(T) layer was expected for the PTB7(T)/PCBM film during the SqD process, which would lead to a further increase in the heterojunction area between PTB7(T) and PCBM. Consequently, the nanostructured PTB7(T) and PCBM layers formed a heterojunction with a large surface area. This result showed that the surface morphology of PTB7 prepared first had an important influence on the heterojunction morphology of the PTB7/PCBM film.

**Figure 3:**
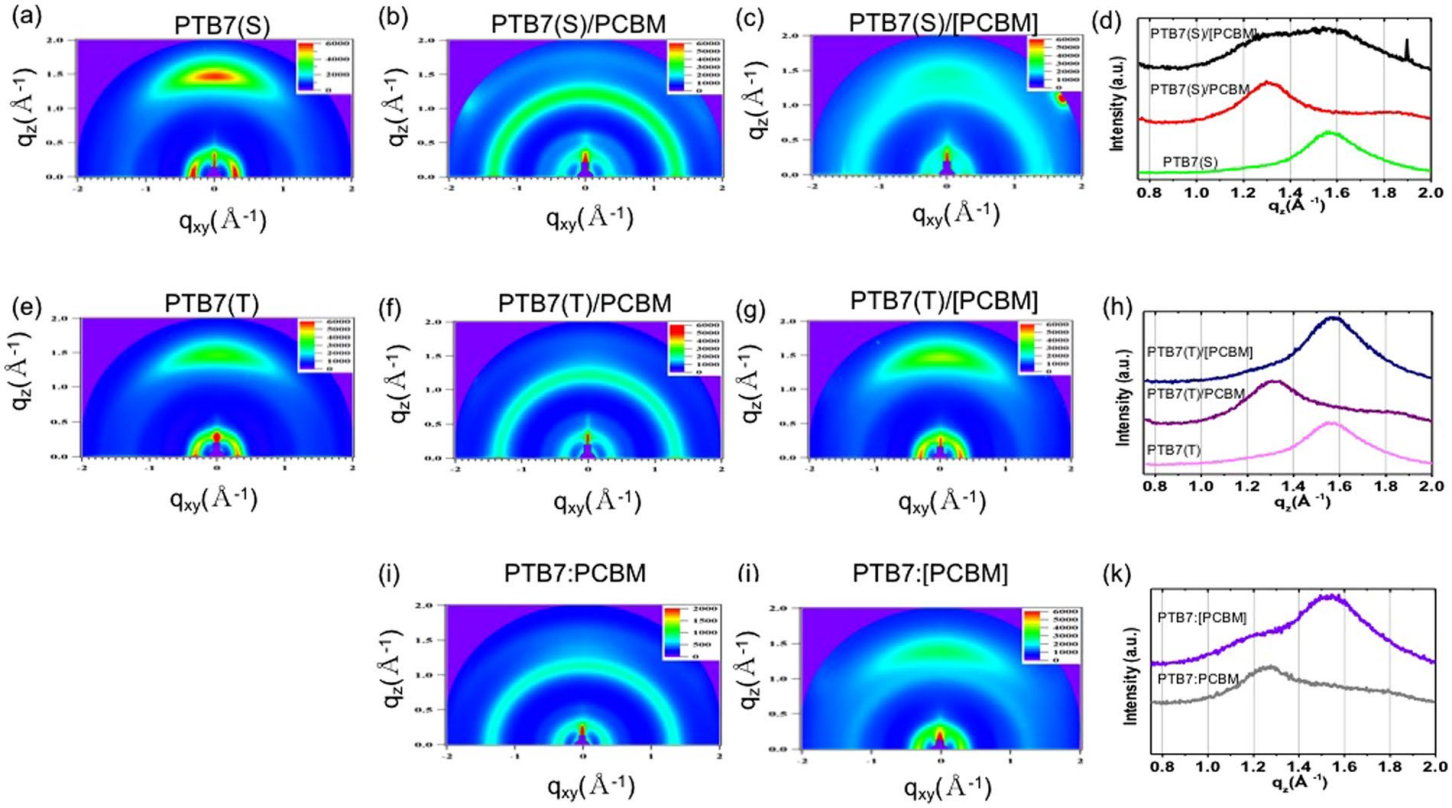
2D GIXRD patterns of (**a**) PTB7(S), (**b**) PTB7(S)/PCBM, (**c**) PTB7(S)/[PCBM], and (**d**) their out-of-plane (*q*_*z*_) line-cuts. 2D GIXRD patterns of (**e**) PTB7(T), (**f**) PTB7(T)/PCBM, (**g**) PTB7(T)/[PCBM], and (**h**) their out-of-plane (*q*_*z*_) line-cuts. 2D GIXRD patterns of (**i**) PTB7:PCBM, (**j**) PTB7(T):[PCBM], and (**k**) their out-of-plane (*q*_*z*_) line-cuts.

The grazing incidence X-ray diffraction (GIXRD) experiments were performed to obtain structural information, such as molecular orientation, intermolecular distances, and crystallite sizes. Figure 3(a)–(h) show the 2D GIXRD patterns and the out-of-plane (*q*_*z*_) scans of PTB7, PTB7/PCBM, and PTB7/[PCBM], respectively. The GIXRD of the BSD film (PTB7:PCBM) and PCBM-removed BSD film (PTB7:[PCBM]) were included in Fig. 3(i)–(k) for comparison. In Fig. 3(a) and (e), both PTB7(S) and PTB7(T) showed a distinct in-plane peak at *q*_*xy*_ = 0.30–0.31 Å^−1^ that corresponded to the (100) Bragg diffraction and the periodic PTB7 lamellae with a spacing of 20.3–20.9 Å. The value was comparable to the cross-sectional diameter of a single PTB7 with a fully extended chain^21^. The out-of-plane peak from the (010) Bragg diffraction at *q*_*z*_ = 1.54–1.56 Å^−1^ was observed from both the PTB7(S) and PTB7(T) films (Fig. 3(a), (d), (e), and (h)). The peak was associated with the π–π stacking spacing of 4.03–4.08 Å. These results suggested that the π-faces of the main chain were approximately parallel to the substrate, and PTB7 had a face-on orientation, which was in agreement with previous reports^22, 23^. The full width at half maximum (FWHM) of the (010) diffraction peak at the q_z_ scan of PTB7(T) was 0.209 Å^−1^, corresponding to the ordered domain size of 4.82 nm. The FWHM value of PTB7(S) was 0.294 Å^−1^, corresponding to the ordered domain size of 3.43 nm. As it was expected, the ordered domain size in PTB7(T) was bigger than that in PTB7(S).

Figure 3(b), (d), (f), and (h) show the 2D GIXRD patterns and *q*_*z*_ scans of PTB7(S)/PCBM and PTB7(T)/PCBM. Both PTB7(T)/PCBM and PTB7(S)/PCBM exhibited a ring-like (100) diffraction at *q* = 0.3–0.4 Å^−1^ from PTB7 and a ring-like (311) diffraction pattern at *q* = 1.32 Å^−1^. The corresponding *q*_*xy*_ scans revealed shifts of the (100) peaks from *q*_*xy*_ = 0.31 Å^−1^ for neat PTB7 to a larger *q* value of 0.35 Å^−1^ for PTB7(T)/PCBM and 0.37 Å^−1^ for PTB7(S)/PCBM. These shifts indicated that the inter-chain spacing of the PTB7 lamella decreased from 20.3 Å to 17.5 Å. In addition, partially overlapped two peaks were observed from the diffraction along *q*_*z*_ (Fig. 3(d) and (h)). These two peaks were resolved by two Gaussian functions centered at *q*_*z*_ = 1.35 ± 0.05 Å^−1^ and *q*_*z*_ = 1.55 ± 0.05 Å^−1^, corresponding to the (311) Bragg, the diffraction of the PCBM^23^, and a π–π stacking spacing of 4.05 ± 0.2 Å between the PTB7 chains, respectively. The relative intensity and the position of the two peaks from the PTB7/PCBM films were almost identical to those from the PTB7:PCBM film prepared through the BSD process (Fig. 3(i) and (k))^21–23^. The (010) diffraction was only observed from the out-of-plane direction. Hence, the PTB7 chains maintained the face-on conformation even after the SqD process.

However, the GIXRD results of PTB7(S)/[PCBM] and PTB7(T)/[PCBM] were significantly different (Fig. 3(c) and (g)). A strong diffraction peak by the PCBM domain was still observed from the PCBM-removed PTB7(S)/PCBM film (PTB7(S)/[PCBM]) (Fig. 3(d)), implying that a significant amount of the PCBM remained in the PTB7(S)/[PCBM] film. The AFM and GIXRD results indicated that PTB7(S) with a relatively low crystallinity was dissolved by the PCBM solvent. A solution containing a mixture of PTB7 and PCBM was then instantaneously formed. Thereafter, phase separation occurred, and the BHJ with a large domain was formed during the solvent evaporation. This BHJ formation process was similar with the BHJ formed by the BSD process utilizing the blended solution of PTB7 and PCBM. However, the AFM results showed that the size of the PTB7 and PCBM aggregations in PTB7(S)/PCBM was significantly bigger than that in PTB7:PCBM prepared through the BSD process because PTB7 had poor solubility for DCM.

It was reported that the pure polymer and PCBM domains and the PTB7 and PCBM intermixture co-existed in the BHJ prepared through the BSD process^13–16^. Similarly, the PTB7(S)/PCBM was expected to have these domain types.

The DIM used for the selective removal of the PCBM was the non-solvent for PTB7. Thus, the DIM would not be able to completely penetrate into the polymer domains. As a result, a complete removal of the PCBM, which existed in the PTB7 and PCBM intermixture, would be difficult. These results indicated existence the intermixture that was formed in PTB7(S)/PCBM fabricated by the SqD process. Comparison of normalized absorption spectra of PTB7(S)/[PCBM] and PTB7(S) support that the PCBM is remained in the PTB7(S)/[PCBM] film (Figure S1(b) in Supporting Information). The similar GIXRD results were observed from the PCBM-removed BSD film (Fig. 3(j) and (k)).

In contrast, the shape and the position of the (010) peak from PTB7(T)/[PCBM] (Fig. 3(g)) was almost identical with that from PTB7(T) (Fig. 3(e)), thereby implying that the PCBM was almost completely removed by the DIM solvent, and the inter-chain distance of PTB7 was recovered to the as-prepared one. The AFM and GIXRD results denote that the BHJ in the PTB7(T)/PCBM film was mainly composed of pure PTB7 and PCBM domains, instead of an intermixture of PTB7 and PCBM.

The intermixed state was not thermodynamically the most stable morphology.^16^ Therefore, a further phase separation was expected to occur when a thermal energy is applied to the film. In other words, the BSD processed film will lose its optimized BHJ morphology under thermal annealing, thereby decreasing the BSD-OPV performance. On the contrary, the PTB7(T)/PCBM composed of less intermixed domains would show a stable performance under thermal annealing.

**Figure 4:**
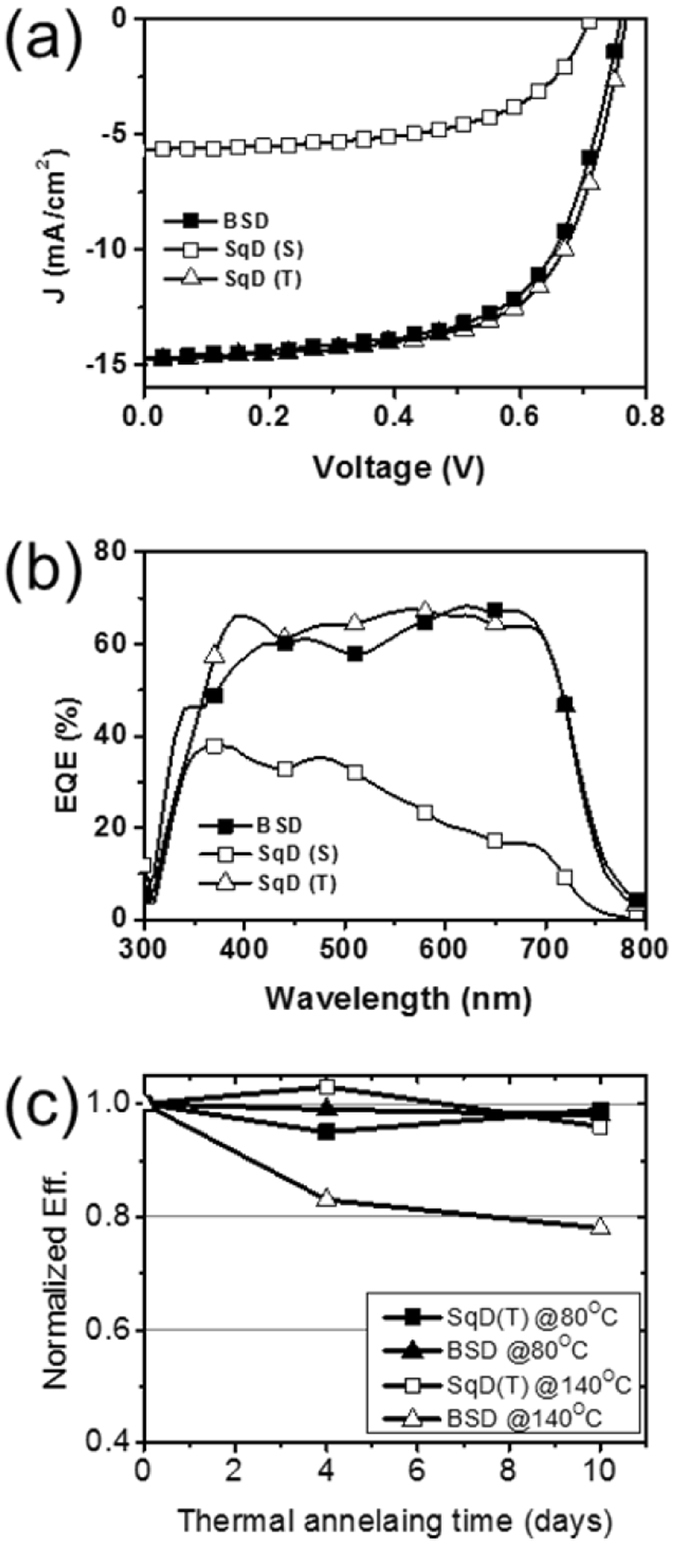
Solar cell performances of BSD, SqD(S), and SqD(T): (**a**) current density–voltage (J–V) curve, (**b**) external quantum efficiency (EQE) spectra, and (**c**) thermal stability test results.

PTB7(S)/PCBM and PTB7(T)/PCBM, which were denoted as SqD(S) and SqD(T), respectively, were utilized to fabricate OPVs and investigate the solar cell performance. Fig. 4(a) shows a comparison of the current density–voltage characteristics of the OPVs, while Table 2 presents their solar cell parameters. As expected from the AFM and GIXRD results, the SqD(T) exhibited an excellent solar cell performance with an open-circuit voltage (*V*_OC_) of 0.768 V, a short-circuit current density (*J*_SC_) of 14.8 mA/cm^2^, a fill factor (*FF*) of 0.654, and a power conversion efficiency (PCE) of 7.43%. The PCE of SqD(T) was significantly higher than SqD(S) (2.33%) and similar with that of the optimized BSD OPV (7.17%). The enhanced PCE of SqD(T) was mainly attributed to the higher *J*_SC_ compared to that of SqD(S). The external quantum efficiency (EQE) spectra of those OPVs were coincidental with the *J*_SC_ results (Fig. 4(b)). For the comparison, the SqD OPV processed with binary solvent containing CB and DIO (SqD(B)) was fabricated and its solar cell performance are shown in Figure S2 and Table S1 in Supporting Information. The PCE of the SqD(B) was obtained to be 6.78%, which was significantly higher than that of the SqD(S), and slightly lower than that of SqD(T). The surface morphology investigation using the AFM (Figure S3 in Supporting Information) revealed that the surface roughness of the PTB7 film was enhanced by processing with the binary solvent. The surface roughness was increased in the order of SqD(S), SqD(B) and SqD(T), which agreed with the tendency of PCE increase.

**Table 2:** Solar cell parameters of the PTB7/PCBM bilayer OPVs

**Device name**	***V***_**OC**_ **(V)**	***J***_**SC**_ **(mA/cm** ^**2**^ **)**	***FF***	***PCE*** **(%)**
BSD	0.760	14.7	0.643	7.17
SqD(S)	0.711	5.68	0.577	2.33
SqD(T)	0.768	14.8	0.654	7.43

Based on the GIXRD and AFM results, the SqD(T) was expected to have better thermal stability than BSD. Many factors other than morphological instability can affect the stability of OPV^24^. Therefore, to exclude influence from other factors, the thermal stability test was performed for the ITO/PEDOT:PSS/photo-active layer. The thermal stability test was conducted at 80 °C for 4 days, at 80 °C for 10 days, at 140 °C for 4 days and at 140 °C for 10 days, respectively. For the control sample, the as-prepared films were stored in the glove box for 10 days at ambient temperature. After completion of the test, films were transferred to vacuum chamber to deposit LiF and Al electrode. As expected, the films processed by SqD(T) exhibited a higher thermal stability compared to the films processed by BSD (Fig. 4(c)). The SqD(T) also exhibited a stable performance for both thermal annealing temperatures. Meanwhile, the PCE of the BSD was stable at the thermal annealing temperature of 80 °C, but decreased to 78% of its initial PCE at the thermal annealing temperature of 140 °C, which was higher than T_g_ of PTB7. This result supported that the BHJ formed by the SqD process was more stable than that formed by the BSD process under thermal stress at high temperature.

In conclusion, an efficient and thermally stable BHJ was prepared by the SqD of PCBM on the nanostructured PTB7 layer. The AFM and GIXRD results revealed that the BHJ formed by the SqD mainly consisted of pure PTB7 and PCBM domains. The OPV utilizing the SqD-processed BHJ exhibited an excellent thermal stability with a comparable efficiency to the OPV utilizing the BSD-processed BHJ.

## Methods

### Film and device fabrication

#### Substrate

Pre-patterned 20 Ω/□ resistive indium tin oxide (ITO) glass substrates were ultrasonically washed in ethyl alcohol, acetone, and isopropyl alcohol for 10 min each. All cells were exposed to UV ozone for 20 min after drying in a convection oven at 80 °C. A PEDOT:PSS (poly(3,4-ethylenedioxythiophene) doped with poly(styrene sulfonate)) (AI4083) solution was vortexed with methanol at a 1:1 ratio. The solution was then deposited onto the substrates by spin coating and dried in a vacuum oven at 110 °C. The final film thickness was approximately 30–35 nm.

### Fabrication of Photoactive layer by SqD process

The poly[4,8-bis[(2-ethylhexyl)oxy]benzo[1,2-b:4,5-b′]dithiophene-2,6-diyl][3-fluoro-2-[(2-thylhexyl)carbonyl]thieno[3,4-b]-thiophenediyl] solution (PTB7, Rieke Metals Inc., USA) was used for the polymer bottom layer. The PTB7(S) solution was prepared by dissolving 14 mg of PTB7 in chlorobenzene (CB). Meanwhile, the PTB7(T) solutions were prepared by dissolving 14 mg of PTB7 in the ternary solvent composed of CB, 1,8-diiodooctane (DIO) and 1-chloronaphthalene (1-CN) (Aldrich, USA). Subsequently, 14 mg of PTB7 was dissolved in 1 ml of 96 vol% CB and 4 vol% DIO, followed by the addition of 1-CN with various volume ratios. The PTB7 solution was spin coated onto the substrate at a speed of 1000 rpm for 15 s, followed by drying at 110 °C in a vacuum oven for 60 min.

A phenyl-C71-butyric-acid-methyl ester (PCBM) (Nano-C, USA) solution (3.25 mg/mL) was prepared in dichloromethane. The PCBM layer was spin coated on top of the PTB7 layer at a speed of 4000 rpm for 10 s. The thickness of PTB7 layer and PTB7/PCBM determined by atomic force microscope were around 80 nm and 90 nm, respectively.

### Fabrication of photoactive layer by BSD

The PTB7:PCBM with weigh ratio of 1:1.5 were dissolved in the solvent composed of chlorobenzene(CB):1,8-Diiodooctane(DIO) with a volume ratio of 96:4. The solution was spin-coated onto the PEDOT:PSS coated substrates at a speed of 1000 rpm for 30 s, followed by drying at 110 °C in a vacuum oven for 60 min.

#### Electrode

Lithium fluoride (LiF) and aluminum (Al) electrodes were deposited via thermal evaporation at a deposition rate of ~0.5 nm/s and a thickness of 100 nm. LiF and Al were thermally evaporated at ~3 × 10^−6^ Torr through a shadow mask. The device area was 0.20 cm^2^.

### Characterization

The current density–voltage characteristics of the OPVs were obtained using the Keithley 2400 SourceMeter under AM 1.5 G irradiation (100 mW/cm^2^) in a 150 W Xenon lamp-based solar simulator (McScience, South Korea) at room temperature.

The external quantum efficiency (EQE) of the OPVs were monochromatically measured by the K3100 EQX IPCE measurement system (McScience, South Korea) using a 300 W Xenon lamp.

The UV–visible absorption spectra were obtained by UV-2450 (Shimadzu, Japan).

The surface topology and the thickness of the films were scanned through atomic force microscopy (AFM) (AFM5100N, Hitachi) in the tapping mode.

The grazing incidence X-ray diffraction (GIXRD) measurements were performed at the PLS-II 9 A U-SAXS beamline of the Pohang Accelerator Laboratory (Korea). The operating conditions were set at a wavelength of 1.12 Å and a sample-to-detector distance of 224 mm. The incidence angle (*α*_*i*_) herein was set at 0.130°. The 2D GIXRD patterns were recorded using a 2D CCD detector (SX-165, Rayonix) with an exposure time of 10–60 s. All the films for the GIXRD had a similar film thickness of ∼80 nm and were spin-coated on PEDOT/PSS-coated Si substrates.

### Thermal stability test

The thermal stability test was conducted for ITO/PEDOT:PSS/SqD(T) and ITO/PEDOT:PSS/BSD films on a hotplate in a nitrogen filled glovebox. Those films were subjected to thermal stability test for two different temperature and two different testing times. In detail, samples were tested at 80 °C for 4 days, at 80 °C for 10 days, at 140 °C for 4 days and at 140 °C for 10 days. For the control sample, the as-prepared ITO/PEDOT:PSS/SqD(T) and ITO/PEDOT:PSS/BSD films were stored in the glove box for 10 days at ambient temperature. After finishing the test, samples were transferred to vacuum chamber to deposit LiF and Al. Since, four samples were used for each condition, total forty devices were tested.

## Electronic supplementary material

Supplementary info
