# Supplementary material for: Formation of Thermally Stable Bulk Heterojunction by Reducing the Polymer and Fullerene Intermixing
Source: Sci Rep. 2017 Aug 29;7:9690. doi: 10.1038/s41598-017-09167-4 (PMC5575051; doi:10.1038/s41598-017-09167-4)
Supplement: Supplementary file 1 — Supplementary info [file 41598_2017_9167_MOESM1_ESM.pdf]

Supporting Information
Formation of Thermally Stable Bulk Heterojunction by Reducing the Polymer and Fullerene Intermixing

Yoonhee Jang[1], Yun Ju Cho[1], Minjung Kim[1], Jeesoo Seok[1], Hyungju Ahn[2], Kyungkon Kim[1*]

[1]Department of Chemistry and Nano Science, Ewha Womans University, Seoul, South Korea

[2] Pohang Accelerator Laboratory, Pohang University of Science and Technology, Pohang 37673, Korea

[*]Corresponding author. Email: kimkk@ewha.ac.kr

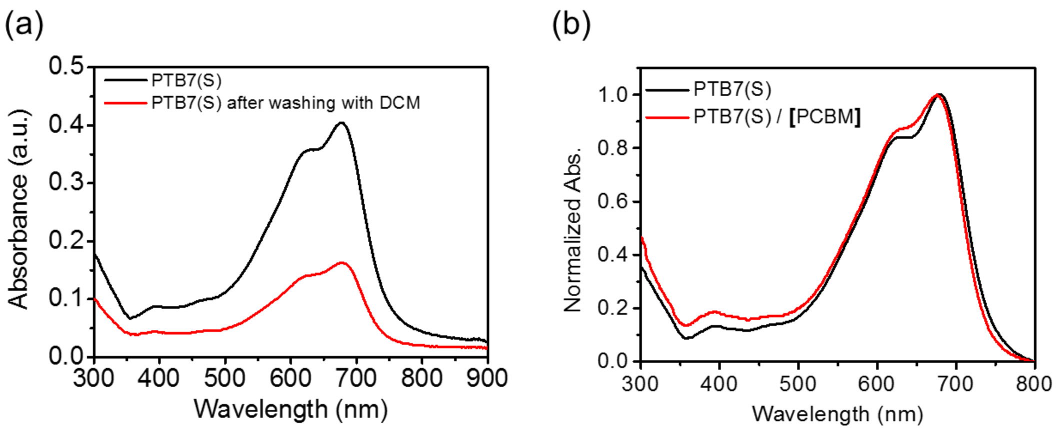

Figure S1. Absorption spectra change of PTB7(S) film (a) after washing with DCM (b) after removal of PCBM from the PTB7(S)/PCBM.

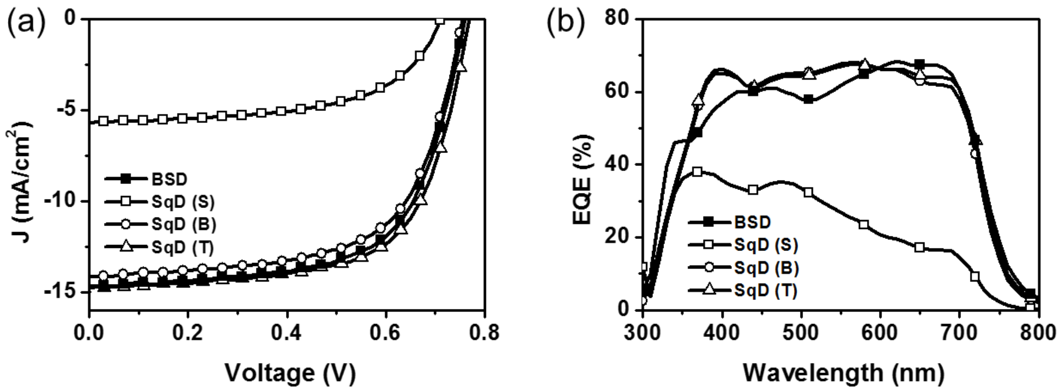

Figure S2. Solar cell performances of BSD, SqD(S), SqD(B) and SqD(T): (a) current density–voltage (J–V) curve, (b) external quantum efficiency (EQE) spectra.

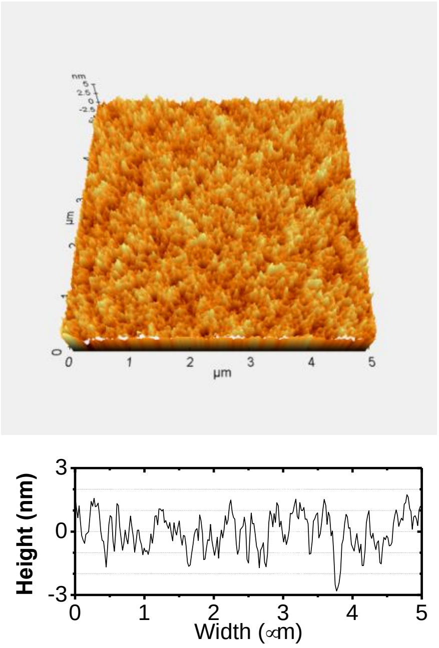

Figure S3. Surface morphology and height profiles of the as prepared PTB7(S) bottom-layer. The image was obtained by AFM in the tapping mode with a scale of 5 × 5 μm^2

Table S1. Solar cell parameters of OPVs

**Device Name**	***V_{OC}* (V)**	***J_{SC}* (mA/cm^2)**	***FF***	***PCE* (%)**
BSD	0.760	14.7	0.643	7.17
SqD(S)	0.711	5.68	0.577	2.33
SqD(B)	0.755	14.1	0.635	6.78
SqD(T)	0.768	14.8	0.654	7.43
